# Practical recommendations for the safe use of gadolinium in magnetic resonance imaging: a Delphi expert panel study

**DOI:** 10.1590/0100-3984.2019.0074

**Published:** 2020

**Authors:** Ronaldo Hueb Baroni, Tufik Bauab Jr., Leonardo Kayat Bittencourt, Giuseppe D’Ippolito, Suzan Menasce Goldman, Guilherme Hohgraefe Neto, Adonis Manzella, Antonio José Rocha, Luis Augusto Sonoda, Fabio Seichi Takeda

**Affiliations:** 1 Instituto Israelita de Ensino e Pesquisa Albert Einstein, Hospital Israelita Albert Einstein, São Paulo, SP, Brazil.; 2 Faculdade de Medicina de São José do Rio Preto (Famerp), São José do Rio Preto, SP, Brazil.; 3 Universidade Federal Fluminense (UFF), Niterói, RJ, Grupo DASA, Rio de Janeiro, RJ, Brazil.; 4 Escola Paulista de Medicina da Universidade Federal de São Paulo (EPM-Unifesp), São Paulo, SP, Brazil.; 5 Hospital Moinhos de Vento, Porto Alegre, RS, Brazil.; 6 Hospital da Restauração, Recife, PE, Centro Diagnóstico Lucilo Ávila Junior, Recife, PE, Brazil.; 7 Faculdade de Ciências Médicas da Santa Casa de São Paulo, São Paulo, SP, Grupo DASA, São Paulo, SP, Brazil.; 8 Instituto de Gastroenterologia de São Paulo (Igesp), São Paulo, SP, Hospital Nipo Brasileiro, São Paulo, SP, Brazil.; 9 Ultramed Medical Services, Londrina, PR, Medvia Diagnósticos, Porto Alegre, RS, Brazil.

**Keywords:** Contrast media, Gadolinium, Magnetic resonance imaging, Gadolinium-based contrast agents, Safety, Meios de contraste, Gadolínio, Ressonância magnética, Agentes de contraste à base de gadolínio, Segurança

## Abstract

**Objective:**

To assess the practical aspects of the use of various gadolinium-based contrast agents (GBCAs) by radiologists.

**Materials and Methods:**

Ten experienced radiologists from different regions of Brazil participated in a Delphi panel querying their use of various GBCAs, including linear and macrocyclic classes (1.0 and 0.5 M), in terms of the choice of agent, volume and dosage of the agents, and associated safety concerns.

**Results:**

The response rate was 100% for all questions. GBCAs are safe in terms of acute adverse reactions, and nephrogenic systemic fibrosis is rare. The deposition of gadolinium in the brain and other tissues is a concern among the experts. Macrocyclic agents are preferable to linear agents; an injection volume below 0.1 mL/kg of a 1.0 M agent could result in good-quality images with additional long-term safety, but there is no published evidence to support this recommendation. The majority of experts preferred not to administer GBCAs to pregnant patients.

**Conclusion:**

When choosing a GBCA, it is important to consider the characteristics of the gadolinium deposition in patient tissues and minimize potential risks. Furthermore, medical education programs are needed to increase the awareness of the potential risks of gadolinium deposition and thus avoid instances of overexposure to the contrast agent.

## Abstract (pt)

**Objetivo:**

Avaliar aspectos práticos do uso de agentes de contraste à base de gadolínio (*gadolinium-based contrast agents* - GBCAs) por médicos especialistas em radiologia.

**Materiais e Métodos:**

Dez radiologistas de diferentes regiões do Brasil foram convidados a participar de um painel Delphi com perguntas sobre o uso de GBCAs lineares e macrocíclicos (1,0 e 0,5 M), em termos de dosagem, volume injetado e preocupações relacionadas à segurança.

**Resultados:**

A taxa de resposta foi de 100% para todas as perguntas. GBCAs são seguros em relação a reações adversas agudas, e os casos de fibrose nefrogênica sistêmica são raros. O depósito de gadolínio no cérebro e em outros tecidos é uma preocupação de todo o painel. Agentes macrocíclicos são preferíveis aos lineares. Um volume menor que 0,1 mL/kg injetado de contraste macrocíclico 1,0 M poderia oferecer imagem com boa qualidade e benefício adicional em longo prazo, entretanto, não há evidências publicadas que apóiem tal recomendação. A maioria prefere não administrar GBCA em gestantes.

**Conclusão:**

É importante considerar as características de deposição e minimizar potenciais riscos ao se escolher um GBCA. Ações de educação médica são necessárias para a conscientização dos potenciais riscos da presença de gadolínio em longo prazo e evitar a sobre-exposição ao agente de contraste.

## INTRODUCTION

Gadolinium-based contrast agents (GBCAs) have been used since 1988, with gadopentate dimeglumine being approved for use in the USA, Germany and Japan^(1)^, and have an excellent overall safety record^(2)^. Acute adverse events are rare^(2,3)^, and the use of newer GBCAs together with careful screening has drastically decreased the incidence of nephrogenic systemic fibrosis^(4)^. However, some concerns remain, such as the deposition of gadolinium in the brain, which was first described in 2014^(5)^. It has been established that linear GBCAs are less stable and show greater deposition in the human brain than macrocyclic agents; deposition also occurs with the latter, albeit to a lesser extent^(6)^. Free gadolinium is highly toxic, and the amount of gadolinium accumulated in tissues is greater for agents with lower stability^(7)^. However, to date, there is no evidence of any damage caused by gadolinium deposition in the brain, and there is no evidence to recommend one class of GBCA over another based on this aspect^(2)^.

Although GBCAs have been considered sufficiently safe, there are differences among the various classes of GBCAs, which include macrocyclic and linear GBCAs. Among the macrocyclic GBCAs are those which contain chelated gadolinium at a high concentration of 1.0 mmol/mL (1 M), such as gadobutrol, and those which contain chelated gadolinium at a concentration of 0.5 mmol/mL (0.5 M), such as gadoteridol and gadoteric acid. Owing to the higher concentration of chelated gadolinium in gadobutrol, a lower volume is required to be administered compared to the volume required for other macrocyclic agents^(8)^.

Owing to the scarcity of published reports addressing the particular characteristics of different GBCAs, radiologists often rely on their personal experience to make decisions in clinical practice.

The Delphi method is a qualitative survey method that allows the collection of opinions and experiences of experts from different geographical areas and makes it possible to deal with complex problems without face-to-face interaction^(9)^. This method is important because it not always possible to conduct randomized clinical trials, which are time and resource consuming^(10^. Therefore, we chose the Delphi method as a feasible way to obtain opinions on a very specific subject from experts in different regions of the country, prioritizing their experience and ensuring that the participants would feel comfortable expressing their thoughts and practices. In addition to the fact that clinical trials are hard to implement, this kind of panel can provide insights and recommendations for future clinical trials with greater focus. Important aspects of the Delphi method are the anonymity of the experts, a defined selection process for the identification of experts, feedback from the experts after each round of questions, a set schedule for obtaining responses from the experts, and participation of at least 10 panelists^(9)^.

In order to support clinical decisions in daily practice, we conducted a Delphi expert panel with radiologists to discuss and share their practices with regard to the use of GBCAs. The aim of this panel was not to reach a consensus, but rather to enrich the discussion on the use of gadolinium in magnetic resonance imaging (MRI), to share the opinions of experts, and thus to help radiologists in their daily practice.

## MATERIALS AND METHODS

Ten radiologists with known experience in MRI were invited to participate in this Delphi panel on the practical issues in the use of GBCAs, excluding organ-specific agents.

The panel initiative originated from the Brazilian affiliate of Bayer, which contracted an independent medical communications agency to assist with survey logistics and question development. All panelists had the opportunity to suggest questions and issues that would be answered by the panel. The panelists answered two web-based anonymous questionnaires sent by the agency; they did not receive any payment to participate in the survey. The questionnaires were sent through a web-based platform (www.allcounted.com). The sponsor did not have any access to the individual answers. The first and second round of questions were sent in February and March 2019, respectively, and the panelists had 10 days to answer each round of questions. The second round included additional questions suggested by the panelists and tie-break questions where there was doubt or conflicting responses. One of the panelists agreed to be the corresponding author and supported the submission process, but apart from this task, all participants contributed equally to this study. The agency generated the first draft of this manuscript, which was then revised and modified by the authors. No ethics committee approval is required for this kind of survey.

## RESULTS

Two women and eight men took part in the panel. Five of them were ≥ 50 years of age, two were between 40 and 49 years of age, and three were between 30 and 39 years of age. All of them had degrees in medicine, had been practicing medicine for more than 10 years, and had a specialist title and/or residency training in radiology.

All questions had a 100% response rate. In order to reflect clinical practice, the panelists were asked to answer according to their experience and opinion, anonymously. The medical communication agency did not access the individual responses.

*- International guidelines and consensus state that macrocyclic GBCAs are safer than linear GBCAs in terms of brain deposition. Do you agree that macrocyclic GBCAs have a lower risk of brain deposition?*

Seven participants answered “yes” and three answered “no”. Two participants said that they had not conducted studies to make such a statement and one said that he/she did not have enough experience with brain MRI.

*- The European Medicines Agency has restricted the use of linear GBCAs, but these are widely used in Brazil. What should be done in Brazil?*

Six participants answered that linear GBCAs should no longer be used, except in cases of organ-specific contrast agents. Four answered that linear GBCAs should continue to be used as a secondary approach.

*- Is gadolinium deposition in body tissues a concern among Brazilian radiologists?*

Six participants answered “yes” and four answered “no”. However, the entire panel is concerned about this potential risk, according to the comments associated with the responses to this question:

- Radiologists outside Brazil care more about this potential risk.
- It should be a cause for concern, but the lack of guidelines and long-term data make it a non-priority. One participant observed that this fact gets more attention outside Brazil.
- The assistant physicians are not concerned with this possibility, or they are unaware of it.

*- What should be done to raise the awareness of the importance of the issue of gadolinium deposition and to decrease the long-term risks?*

**Figure 1:**
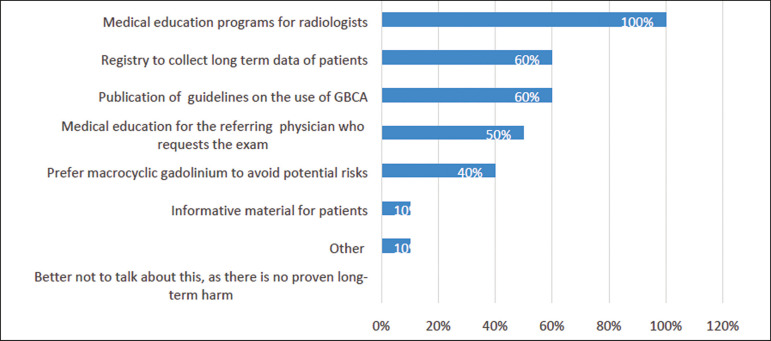
Recommendations to raise awareness of gadolinium deposition and decrease the potential associated long-term risks.

The participants could choose any alternative that was applicable. The answers are summarized in Figure 1.

The “other” recommendation was dose reduction.

*- Is there a difference in the image resolution obtained on using 1.0 M (1 mol/L) gadolinium and that obtained using 0.5 M gadolinium?*

Two participants answered “yes”, four answered “no”, and four answered “other”. The “other” responses were explained as follows:

- In some cases, 1.0 M gadolinium results in superior image resolution.
- It depends on the volume to be injected.
- There are differences between gadobutrol and gadoteric acid.
- Theoretically, 1.0 M gadolinium could result in a superior image. However, I do not see it in my practice.

*- Do you think that, ideally, we should reduce the volume of 1.0 M GBCA used, in order to adjust it to the lowest effective dosage that enables the obtainment of a good-quality image?*

Nine participants answered “yes, we should ideally reduce the injected volume”. One participant commented that this reduction should be done only in case of renal impairment. Overall, they stated that their opinion was based on an ideal situation, but evidence is needed to make such statement.

The same question was posed regarding the volume of 0.5 M GBCA. Again, the majority (eight participants) agreed that ideally the volume should be reduced whenever possible.

One participant reminded that image quality correlates with dosage, but in cases of exams of the pancreas and liver, it is not possible to reduce the dosage.

In this question, we asked the radiologists whether the volume should be ideally reduced. In the next question, we asked if it was feasible.

*- Is it possible to use 1.0 M GBCA at dosages/volumes lower than 0.1 mL/kg and still obtain an image with good accuracy for diagnosis?*

We received seven “yes” and three “no” responses. One participant confirmed that he/she had obtained good results with lower dosages according to personal experience; another observed that reducing the dose would be adequate in some cases, but not for the pancreas and liver.

*- Does the minimal volume to be injected depend on the scanner and protocol?*

Seven participants answered “yes”, two answered “no”, and one said that it also depended on the clinical condition of the patient.

*- What is your perception of the image quality obtained with 1.0 M GBCA in dosages lower than 0.1 mL/kg?*

Seven participants answered that they perceived the image quality to be at least as good as that obtained with the dosage of 0.1 mL/kg. The words used for these answers were “excellent”, “satisfactory”, “good”, and “the same”. This was not a multiple-choice question. One participant answered that the quality was worse, and two said that they did not have experience with this dosage.

We had one more discussion about this perception on the second round of questions; overall, the participants reaffirmed that this was a perception, and more evidence is needed before confirming that a lower dose is efficient. Some participants were concerned that a dosage below 0.1 mL/kg was an “off-label” dosage. However, the prescribing information states that 0.1 mL/kg is “usually sufficient”, but does not state that is mandatory to use this dosage^(11)^.

Six participants reported that they had experience with a lower dosage of 1.0 M GBCA.

*- Is it possible to make an accurate diagnosis using lower dosages of 0.5 M GBCA? What is your opinion?*

Five participants answered that it was possible to make a good diagnosis with a lower dosage of 0.5 M GBCA. Those who disagreed justified their responses with a lack of experience with a lower dosage and a lack of evidence.

*- Considering the last reports of brain deposition, although we do not know the long-term effects of gadolinium deposition in the brain, do you think it is a concern that could justify the use of a lower volume of 1.0 M GBCA?*

Nine answered “yes”.

*- In your opinion, what would be the minimal dosage of 1.0 M GBCA and 0.5 M GBCA that would allow for an accurate diagnosis?*

Four participants gave their opinion on a minimal dosage below 0.1 mL/kg for 1.0 M GBCA and 0.2 mL/kg for 0.5 M GBCA. For 1.0 M GBCA, one suggested a dosage of 0.035 mL/kg and another participant suggested a dosage of 0.06 mL/kg. For 0.5 M GBCA, one participant suggested a dosage of 0.15 mL/kg. One participant estimated an approximately 20% reduction and another participant mentioned that the experimental use of deep learning would allow a reduction to 10% of the current dosage. The participants also said that evidence is necessary before any recommendation, and it is important to consider the body region that is being examined.

*- Do you believe that the lower injected volume decreases the risk for acute adverse reactions (e.g., allergic reactions)?*

Nine participants answered “no” to this question, and there was an agreement that the acute reactions do not depend on the dosage. However, one participant observed that although the dosage is not a risk factor for acute reactions, a lower dosage might be important to prevent nephrogenic systemic fibrosis and gadolinium deposition in tissues.

*- Are macrocyclic GBCAs (0.5 M and 1.0 M) safe to be used in children, including those younger than 2 years in age and neonates?*

Seven participants agreed that macrocyclic GBCAs are safe for use in children, and three participants made observations agreeing with the safety of GBCAs. One mentioned a lack of experience with children, and two others recommended GBCA use only if extremely necessary, as its approval for use in children younger than 1 year in age is more recent and the urinary system in such children is immature.

*- Is it necessary to adjust the dosage/volume (mL/kg) in children?*

Nine participants answered “no” to this question.

*- Are macrocyclic agents safer than linear agents, in any age group?*

Eight participants answered “yes”, and two answered “no”. Two participants observed that macrocyclic agents are safer in terms of deposition in the brain and other tissues, but not in terms of acute adverse reactions.

*- GBCAs should not be used in patients with severe renal impairment (glomerular filtration rate < 30 mL/min).*

Although seven participants agreed with this statement and three disagreed, the participants observed that it could be done if necessary, as justified by the clinical need. In such cases, a macrocyclic agent should be used.

*- What should be done when the patient has impaired renal function, but this impairment is not severe (glomerular filtration rate > 30 mL/min)?*

**Figure 2:**
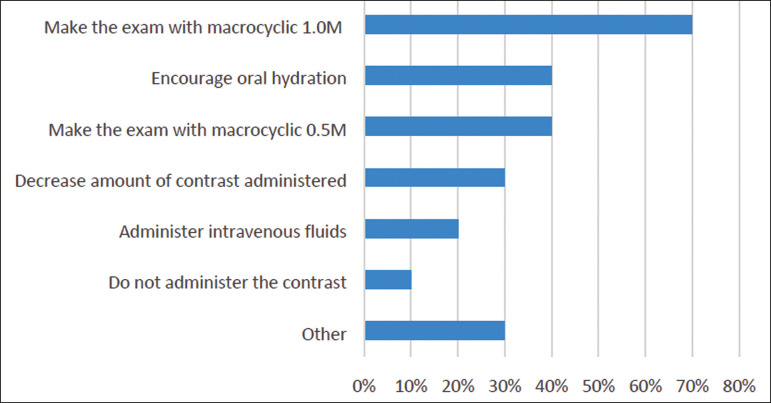
Management of patients with mild to moderate renal impairment.

In this question, multiple choices were accepted. The answers are summarized in Figure 2. The “other” answers were comments added to the alternatives:

- Two participants added: “It depends on the creatinine clearance”.
- One participant commented that he/she would first check for the real need of contrast.

*- In your experience, do you think there is an anatomical region where a dosage/volume of 1.0 M macrocyclic GBCA lower than 0.1 mL/kg could provide an image with good accuracy?*

Six answered “do not know”, and four gave their opinion, as follows:

- Possibly any region, including the heart.
- Pelvis, abdomen, cranial and joints.
- Musculoskeletal.
- Brain.

*- In your experience, are there differences between 1.0 M GBCA and 0.5 M GBCA in terms of artifacts?*

Only one participant observed differences in the presence of artifacts, observing that artifacts occur with gadobutrol in the pancreas, liver, and kidneys. Another panelist stated that it is theoretically possible to have artifacts with gadobutrol owing to its higher concentration; however, he/she did not observe it in clinical practice.

*- Does the use of high-relaxivity contrast agents allow for sequences with better spatial resolution?*

Half of our panel agreed that higher relaxivity allows for better spatial resolution. One participant observed that the statement is correct if the aim is to investigate whether the production of more signal can aid in the detection of lesions. One participant reminded that in MRI, the concept of spatial resolution is related to the voxel size, which is a consequence of the slice thickness, field of view, and matrix size.

*- In a non-emergency setting, would you administer GBCAs to a pregnant patient or to a patient with a suspicion of pregnancy?*

**Figure 3:**
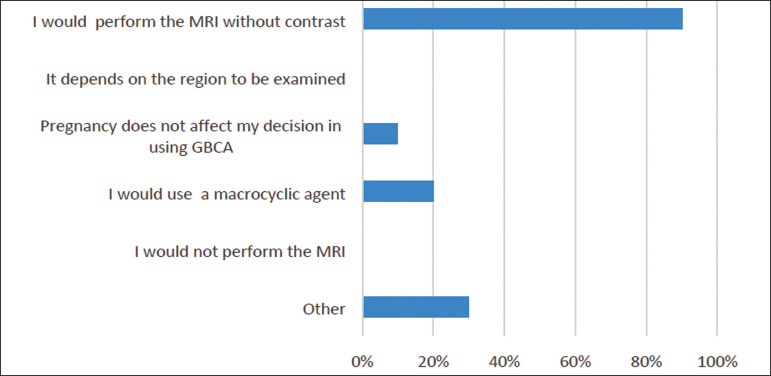
Use of GBCAs in a pregnant patient in a non-emergency setting.

Multiple choices were accepted. The answers are summarized in Figure 3.

Again, it is important to weigh the risks and benefits, but it is preferable to not to use GBCAs in pregnant patients; however, if there is a real need for contrast, the use of a macrocyclic agent rather than a linear agent would be advisable.

*- Would you administer GBCA to a woman who is breastfeeding?*

**Figure 4:**
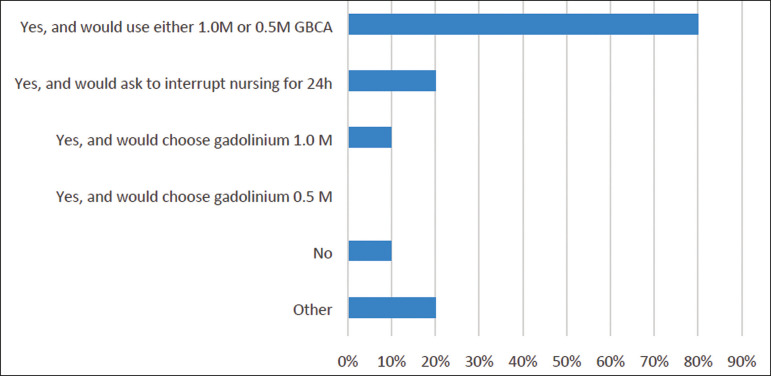
Use of GBCAs in women who are breastfeeding.

Multiple choices were accepted. Results are summarized in Figure 4.

One participant observed that a linear agent could be used. Also, if the patient is concerned about any risks, she could be asked to store breast milk before the exam and refrain from breastfeeding for 24 hours after the exam.

*- Which criteria should be adopted for the diagnosis of acute renal insufficiency in clinical practice?*

Eight participants answered that the European Society of Urogenital Radiology criteria should be used, one participant preferred the Kidney Disease Improving Global Outcomes criteria, and one participant answered “both”.

## DISCUSSION

Radiologists have long been using GBCAs in their daily practice, often without clear recommendations or evidence regarding the differences among the agents. Although some studies suggest that linear GBCAs deposit more gadolinium in the brain than macrocyclic GBCAs^(6,12-14)^, linear GBCAs are still widely used in Brazil. Our panel tended to agree that the use of linear agents should be restricted to organ-specific exams (60% agreement) or as a secondary approach (40% agreement).

There are also differences among macrocyclic agents. Half of our panel believed that it is possible to use a lower volume of 1.0 M GBCA to produce good-quality images, and nine experts (90%) believe the injected volume should be ideally reduced, in order to avoid the potential risks of the gadolinium deposition in the brain, bones, and skin. However, despite the experiences reported by members of the panel, the lack of controlled studies using lower volumes of gadolinium, either 0.5 M or 1.0 M, is a concern. The panelists suggested that clinical studies with lower volumes of gadolinium are necessary.

The panel discussed issues related to pregnancy, breastfeeding, infants, and renal impairment; there were concerns regarding the potential unknown impact of gadolinium deposition and the lack of evidence to support several decisions.

All experts agreed that a medical education program for specialists could raise awareness of the issues related to clinical decisions and long-term outcomes.

The strength of this Delphi panel was the undeniable expertise of the panelists. The limitations of this panel were the low number of participants and the fact that its findings are based on expert opinion and not evidence from clinical studies.

The aim of this panel was not to reach a consensus, but rather to enrich the discussion on the use of gadolinium in MRI, to share the opinion of experts, and thus to help radiologists in their daily practice.

## CONCLUSION

It is the opinion of the panel that it is important to consider characteristics of gadolinium deposition in patient tissues and to minimize potential short and long-term risks when choosing a GBCA. Caution is required with pregnant and breastfeeding women, people with renal impairment, and children younger than 1 year in age.
